# Variability in Genomic and Virulent Properties of *Porphyromonas gingivalis* Strains Isolated From Healthy and Severe Chronic Periodontitis Individuals

**DOI:** 10.3389/fcimb.2019.00246

**Published:** 2019-07-10

**Authors:** Katterinne N. Mendez, Anilei Hoare, Cristopher Soto, Isaac Bugueño, Marcela Olivera, Claudio Meneses, Jose Manuel Pérez-Donoso, Eduardo Castro-Nallar, Denisse Bravo

**Affiliations:** ^1^Center for Bioinformatics and Integrative Biology, Facultad de Ciencias de la Vida, Universidad Andrés Bello, Santiago, Chile; ^2^Laboratorio de Microbiología Oral, Facultad de Odontología, Universidad de Chile, Santiago, Chile; ^3^Centro de Biotecnología Vegetal, Facultad de Ciencias de la Vida, Universidad Andrés Bello, Santiago, Chile; ^4^FONDAP Center for Genome Regulation, Santiago, Chile

**Keywords:** *Porphyromonas gingivalis*, periodontitis, comparative genomics, hemagglutination, fimbrium, biofilm

## Abstract

*Porphyromonas gingivalis* has been extensively associated with both the onset and progression of periodontitis. We previously isolated and characterized two *P. gingivalis* strains, one from a patient exhibiting severe chronic periodontitis (CP3) and another from a periodontally healthy individual (H3). We previously showed that CP3 and H3 exhibit differences in virulence since H3 showed a lower resistance to cationic peptides compared with CP3, and a lower ability to induce proliferation in gingival epithelial cells. Here, we aimed to determine whether differences in virulence between these two strains are associated with the presence or absence of specific genes encoding virulence factors. We sequenced the whole genomes of both *P. gingivalis* CP3 and H3 and conducted a comparative analysis regarding *P. gingivalis* virulence genetic determinants. To do so, we performed a homology search of predicted protein sequences in CP3 and H3 genomes against the most characterized virulence genes for *P. gingivalis* available in the literature. In addition, we performed a genomic comparison of CP3 and H3 with all the 62 genomes of *P. gingivalis* found in NCBI's RefSeq database. This approach allowed us to determine the evolutionary relationships of CP3 and H3 with other virulent and avirulent strains; and additionally, to detect variability in presence/absence of virulence genes among *P. gingivalis* genomes. Our results show genetic variability in the hemagglutinin genes. While CP3 possesses one copy of *hagA* and two of *hagC*, H3 has no *hagA* and only one copy of *hagC*. Experimentally, this finding is related to lower *in vitro* hemmaglutination ability of H3 compared to CP3. Moreover, while CP3 encodes a gene for a major fimbrium subunit FimA type 4 (CP3_00160), H3 possess a FimA type 1 (H3_01400). Such genetic differences are in agreement with both lower biofilm formation ability and less intracellular invasion to oral epithelial cells exhibited by H3, compared with the virulent strain CP3. Therefore, here we provide new results on the genome sequences, comparative genomics analyses, and phenotypic analyses of two *P. gingivalis* strains. The genomics comparison of these two strains with the other 62 genomes included in the analysis provided relevant results regarding genetic determinants and their association with *P. gingivalis* virulence.

## Introduction

*Porphyromonas gingivalis* is a Gram-negative anaerobic bacterium that significantly contributes to the onset and progression of periodontitis (Hajishengallis, 2015), an oral disease affecting 30–50% worldwide adult population (Petersen and Ogawa, 2012). Besides, *P. gingivalis* has also been associated with increased risk of developing other prevalent diseases, such as cardiovascular diseases, rheumatoid arthritis, metabolic diseases, cancer, and Alzheimer's disease, among others (Babic et al., 2015; Kumar, 2017).

*P. gingivalis* has been proposed to be a keystone pathogen in the etiology of periodontitis, since it can promote changes in homeostasis of the commensal microbiota, contributing to the ecological successions occurring in the subgingival area that are associated with inflammation and tissue destruction in *in vivo* models (Hajishengallis et al., 2011; Hajishengallis, 2015). Nonetheless, *P. gingivalis* is present in the subgingival biofilm of both diseased subjects and periodontally healthy individuals, although the frequency of detection is higher in the first group (Lau et al., 2004; Kumawat et al., 2016; Kulkarni et al., 2018). From an ecologic point of view, this species is classified as a pathobiont, being naturally part of the subgingival microbiota in periodontally healthy individuals but, under certain environmental conditions, contributing to or exacerbating the disease state (Hajishengallis and Lamont, 2012; Cugini et al., 2013).

Interestingly, *P. gingivalis* has not only been found in higher loads in the subgingival biofilm in periodontitis subjects compared to healthy individuals, their virulent properties vary among strains isolated from each condition. In previous studies, we reported that clinical isolates of *P. gingivalis* obtained from healthy (H1, H2, and H3) and periodontitis subjects (CP1, CP2, CP3) show differences in their virulence. For example, strains from periodontitis patients exhibit a higher resistance to antimicrobial peptides (Díaz et al., 2015) and higher ability to decrease apoptosis of host cells and therefore, to persist intracellularly (Soto et al., 2016). Among them, CP3 and H3 respectively, showed the highest and lowest resistance to antimicrobial peptides and ability to both decrease apoptosis and persist intracellularly, respectively. In agreement with these results, CP3 was obtained from a subject showing the most severe clinical signs of periodontitis, while the subject that donated the sample containing H3, showed to be periodontally healthy.

Several virulence factors contribute to differences in the bacterial virulence of *P. gingivalis*, in particular the Fimbriae and hemagglutinins. Based on the diversity of the *fimA* gene, fimbriae are classified into six genotypes (I-V and Ib) (Hamada et al., 1994; Nakagawa et al., 2000), being type II fimbriae, followed by type IV, the most frequently found in *P. gingivalis* strains isolated from periodontitis patients (Amano et al., 2000, 2004; Missailidis et al., 2004). In contrast, healthy adults carry strains coding type I *fimA* (Amano et al., 2000). This is not surprising since strains expressing type II FimA have been associated with both higher abilities to adhere and invade epithelial cells (Nakagawa et al., 2002; Amano et al., 2004), as well as an increased production of biofilm (Kuboniwa et al., 2009). Particularly, while strains harboring type II and IV FimA produce biofilms with significantly higher biovolumens with clumped and tall colonies, type I FimA strains form biofilms with a dense basal monolayer and disperse microcolonies (Kuboniwa et al., 2009).

Hemagglutinins are surface proteins associated with bacterial adhesion to the host cells and to erythrocyte agglutination. These proteins facilitate the acquisition of nutrients (heme) by the bacterium through erythrocyte binding (Lépine and Progulske-Fox, 1996). *P. gingivalis* possesses at least eight hemagglutinins encoded by the *hag* genes (*hagA* to *hagE*) (Progulske-Fox et al., 1989, 1995; Nelson et al., 2003). Among them, the hemagglutinins HagA, HagB, and HagC have been described as important virulence factors (Bélanger et al., 2012; Connolly et al., 2017). HagA is involved in adhesion to both gingival epithelial and endothelial cells (Bélanger et al., 2012); as well as contributing to bacterial survival in both epithelial cells *in vitro* and murine abscesses (Miller et al., 2017). Conversely, HagB and HagC do not contribute to *P. gingivalis* virulence *in vivo* (Miller et al., 2017), although HagB has been associated with bacterial adhesion *in vitro* (Song et al., 2005).

Virulent and avirulent *P. gingivalis* strains have been compared in previous studies in order to find genetic determinants that explain the differences in virulence. The comparison between the virulent strain W83 of *P. gingivalis* with the attenuated strain 33277, using microarrays, showed that their chromosomes were very similar, with ~93% of the predicted genes in common. Intriguingly, 7% of the genes showed very low or no signals in ATCC 33277, suggesting that the ORFs analyzed are absent or divergent in 33277 strain (Chen et al., 2004). One of the most important differences found was the presence of an ORF involved in the synthesis of capsular polysaccharide in W83, which was absent in 33277 (Chen et al., 2004). This is an important virulence factor that contributes to inflammation and participate in the interaction of *P. gingivalis* with other members of the oral biofilm (Laine and van Winkelhoff, 1998; Davey and Duncan, 2006). Moreover, by using comparative genomics, extensive genomic rearrangements were observed in W83 compared with 33277, including inversions, translocations, deletions or replacements (Naito et al., 2008). More directly associated with pathogenic mechanisms, a genomic comparison between an invasive (W83) and a non-invasive (AJW4) strain of *P. gingivalis* was performed using whole genome microarray, showing a series of hypothetical ORF, which are polymorphic (Dolgilevich et al., 2011). Moreover, some of them were associated to the invasion of endothelial cells *in vitro*, explaining –at least in part- why the AJW4 strain is non-invasive.

In this work, we performed whole genome sequencing of a hyper virulent strain (CP3) obtained from a Chilean periodontitis patient exhibiting severe clinical signs of inflammation and tissue damage, as well as of an attenuated strain (H3) obtained from a periodontally-healthy individual. In addition, we performed a comparison between CP3 and H3 with all the available genomes of *P. gingivalis* through whole-genome nucleotide identity and a maximum likelihood phylogenetic inference to resolve the evolutionary relationships among *P. gingivalis* strains. Using pan-genome analysis and homology searches, we evaluated the presence and absence of genes associated with virulence factors in order to test for differences in gene content contributing to bacterial virulence. We next evaluated whether such differences correlated with different virulence properties *in vitro*, by performing gingival epithelial cell adhesion/invasion assays, as well as biofilm and hemagglutination experiments *in vitro*.

## Materials and Methods

### Strain Information

CP3 strain was isolated from a subgingival plaque pool of a patient with severe periodontitis, while H3 strain was isolated from a periodontally healthy subject at the Facultad de Odontología, Universidad de Chile. Periodontal status was evaluated by a calibrated clinician considering clinical parameters of periodontitis severity, such as clinical attachment level (CAL) and probing depth (PD) (Table S1). While CP3, showed high virulent properties *in vitro* even when compared to the highly virulent strain W50; H3 showed low virulent properties *in vitro* (Díaz et al., 2015; Soto et al., 2016)

### Bacterial Strains and Culture Conditions

*P. gingivalis* reference strains ATCC 33277, W50 (ATCC 53978), and clinical isolates CP3 and H3, were grown as described previously (Soto et al., 2016). Briefly, they were grown in an anaerobic atmosphere at 37°C in blood agar (5% defibrinated sheep blood) or in brain-heart infusion broth (BHI; Oxoid), both supplemented with 5 mg/mL hemin-menadione.

### Invasion and Adhesion Assays to Gingival Epithelial Cells

*P. gingivalis* was grown in BHI supplemented with 5 mg/mL hemin-menadione and incubated at 37°C in anaerobiosis to an optical density of 600 nm (OD_600_) = 0.6–0.8. For invasion assays, gingival epithelial cells (OKF6/TERT2) were infected at a multiplicity of infection (MOI) of 100 for 90 min at 37°C and 5% CO_2_. After infection, cells were washed with PBS and incubated 2 h with fresh media supplemented with 300 ug/mL gentamicin and 200 μg/mL metronidazole. Then, cells were rinsed with PBS and incubated with 1% saponin, followed by serial dilution of the resulting supernatant, and plating on blood agar plates supplemented with hemin and menadione. After 5–7 days, colony-forming units (CFU) were quantified. Adhesion assays were performed as described above, except that cells were infected at 4°C. Results were compared by applying ANOVA testing and Dunnett's multiple comparison to analyze statistical differences.

### DNA Sequencing and Genome Assembly

Total DNA was extracted from the *P. gingivalis* clinical isolates CP3 and H3 in tubes containing lysis/stabilization buffer. Samples were lysed using bead-beating, and DNA was extracted by a guanidine thiocyanate silica column-based purification method (Almonacid et al., 2017). CP3 and H3 DNA samples were used to prepare paired-end libraries following the Illumina sample preparation guide and sequenced in an Illumina MiSeq at the Centro de Biotecnología Vegetal, Universidad Andrés Bello (Santiago, Chile). For the CP3 sample, two DNA libraries, “L1” and “L2,” were prepared and sequenced (technical replicate). For quality control, reads were screened and filtered for adapter sequences and reads shorter than 50 bp. Reads with low quality bases (<Q20) were trimmed using FastQC v0.11.8 and PRINSEQ v.0.20.4 (Andrews, 2010; Schmieder and Edwards, 2011). Quality-controlled reads of CP3 libraries L1 and L2, and H3 library were used to perform a *de novo* assembly of both CP3 and H3 genomes, using SPAdes v3.10.0 (Bankevich et al., 2012). The assembled genomes were annotated using Prokka v1.12 (Seemann, 2014). CP3 and H3 genome assemblies were evaluated by the calculation of statistical values (e.g., N50, number of contigs and bases, and GC content) and completeness analysis through the search of bacterial ortholog genes (OrthoDB database), using NGSQCToolkit v2.3.3 and BUSCO v3 (Patel and Jain, 2012; Waterhouse et al., 2017), respectively. Also, quality-controlled reads were aligned against the assembled contigs to calculate CP3 and H3 genomes coverage, using Bowtie v 2.3.4.1 and Samtools v1.7 (Li et al., 2009; Langmead and Salzberg, 2012), respectively.

### *P. gingivalis* Genomic Dataset

In order to perform a comparative genomic analysis of CP3 and H3 with others *P. gingivalis* strains, a total of 62 *P. gingivalis* genomes were retrieved from NCBI, RefSeq database (as of February 2018). All downloaded *P. gingivalis* genomes were reannotated using Prokka as with CP3 and H3 genomes.

### Whole-Genome Nucleotide Identity and Phylogenetic Relationships

Average nucleotide identity (ANI) was calculated for all 64-genome *P. gingivalis* dataset (i.e., 62 genomes from RefSeq plus CP3 and H3 genomes), using the pyani Python3 module (Pritchard et al., 2016) and pheatmap R packages (https://cran.r-project.org/web/packages/pheatmap/index.html) for results' visualization. kSNP3.0 was used for SNP identification in the 64 *P. gingivalis* genomes and the core polymorphic positions were used for the phylogenetic reconstruction through Maximum Likelihood inference using RAxML v8.2.9. The obtained tree was plotted using FigTree v1.4.4 (Stamatakis, 2014; Gardner et al., 2015) (http://tree.bio.ed.ac.uk/software/figtree/).

### Pan-Genome Analysis and Search for Virulence Genes

The pan-genome analysis of the 64-genome *P. gingivalis* was performed through the Roary v3.7.0 pipeline (Page et al., 2015). For virulence gene identification, a homology search of predicted protein sequences in CP3 and H3 genomes against the most characterized virulence genes identified in *P. gingivalis* available in the literature was performed, using CRB-Blast v0.6.6 (https://github.com/cboursnell/crb-blast). Also, virulence genes were identified in the 64-genome dataset using pan-genome results to evaluate presence/absence and protein sequence variation of these genes. According to shared and unique genes between CP3 and H3 genomes, a Venn diagram was plotted using the VennDiagram R package (https://cran.r-project.org/package=VennDiagram). The inferred phylogenetic tree was annotated based on the presence/absence of the virulence genes that are part of the accessory genome, using the pheatmap R package and FigTree.

### Biofilm Formation Assays

*P. gingivalis* cultures grown to exponential phase were obtained, diluted, and inoculated to each well of a 96-well flat bottom plate in supplemented BHI medium (and 1% tryptic soy broth, TSB). Then, plates were incubated anaerobically at 37°C for 72 h. After the incubation time, the supernatant was removed, wells were washed twice by immersion in distilled water and the plate was left to air dry for 1 h. Biofilm staining was performed by adding 100 μL of 0.1% safranin and incubating for 15 min, followed by two washes by immersion in distilled water. Finally, the dye retained in the biofilm was eluted incubating with 100 μL of 95% ethanol for 5 min, the elution was transferred to a new 96-well plate and the absorbance was measured at 490 nm. Results were compared by applying ANOVA testing and Dunnett's multiple comparison to analyze statistical differences.

### Hemagglutination Assays

One mL of defibrinated horse blood was centrifuged at 2,100 rpm for 5 min. The resulting pellet (red blood cells) was washed 3 times and then diluted in PBS to 2% solution. In parallel, *P. gingivalis* strains (W50, H3, and CP3) were grown to an exponential phase culture, which was adjusted to DO_600_ = 2.0 (3 × 10^8^ CFU/mL). Then, two hundred μL of each suspension was added to one well of a 96-well round-bottom plate. After that, each suspension (W50, H3, and CP3) was serially diluted, by taking 100 μL and mixed with 100 μL PBS (1:2 dilution). This step was repeated until 1:64 dilution. Finally, each well was mixed with an equal volume of 2% sheep erythrocytes at 37°C for 3 h. Experiments were repeated three times.

### *hagC* Expression Analysis by Quantitative Reverse Transcription Polymerase Chain Reaction

Total cytoplasmic RNA was isolated from CP3, W50, and H3 strains using the TRIZOL method as described previously (Chomczynski and Mackey, 1995). Reverse transcription was performed using M-MLV reverse transcriptase (Promega, Madison, WI, USA). To quantify the mRNA expression for *hagC* and 16S rRNA genes, 1,000 ng of RNA were used to synthetize cDNA and amplify by quantitative real-time polymerase chain reaction (qRT-PCR) using appropriate primers (HagC: TTTGCCAAGAATGTGCTGAC and GTCGAGGGCTATGACCTGAG; 16S: AGGCAGCTTGCCATACTGCG and ACTGTTAGCAACTACCGATGT) and the Brilliant II SYBR qPCR master mix (Agilent, Santa Clara, CA, USA) in a Mx3000p qPCR system (Agilent, Santa Clara, CA, USA). The cycling program was as follows: 95°C for 10 min, followed by 45 cycles of 95°C for 10 s, 58°C for 30 s, and 72°C for 20 s. Finally, a melting curve was obtained by incubating at 95°C for 15 s, 60°C for 1 min, and 95°C for 15 s, to detect non-specific product formation and false positive amplification. As an endogenous control, we determined the16S rRNA gene expression levels. Data were statistically analyzed using PRISM software (version 6.0; GraphPad, La Jolla, CA, USA). Comparisons between strains were made using by applying ANOVA testing and Dunnett's multiple comparison to analyze statistical differences.

### Data Availability

This Whole Genome Shotgun project has been deposited at DDBJ/ENA/GenBank under the accession SGBA00000000 (CP3) and SGAZ00000000 (H3), under BioProject PRJNA521311. The version described in this paper is version SGBA01000000 (CP3) and SGAZ01000000 (H3).

## Results

### *P. gingivalis* CP3 and H3 Genomes

A total of 3.43 million sequences were sequenced and an average of 8% of the raw sequence reads were filtered out. Quality-controlled reads of the CP3 L1 and L2 libraries, and H3 library were used to perform the *de novo* assembly of the 2.25 and 2.31 Mb-length genomes of CP3 and H3, respectively. CP3 and H3 genomes are composed by 118 and 165 contigs, with a N50 value of 37,600 and 39,176 bp, GC content of 48.47 and 48.35%, and predicted protein count of 1,896 and 1,955, respectively. These values are similar with average statistics values for the 62 *P. gingivalis* available genomes at NCBI's RefSeq database (length 2.33 Mb, protein count 1,896, and GC content 48.4%; as of February 2018). Both assembled genomes exhibit high completeness, having a 95.3% of bacterial orthologs present in the OrthoDB database. The average coverage of CP3 and H3 genomes is 300x and 28x, respectively. Genome features of both strains are displayed in Table S2.

### Whole-Genome Nucleotide Identity and Phylogenetic Relationships of *P. gingivalis* Strains

**Figure 1:**
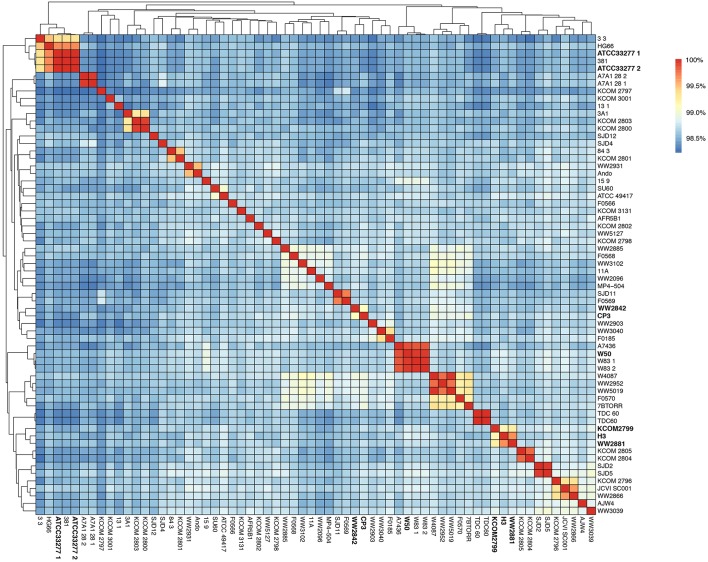
Average nucleotide identity (ANI) in the 64-genome *Porphyromonas gingivalis* dataset. Hierarchical clustering of the *P. gingivalis* genomes based on their average nucleotide identity values. The color gradient from blue to red shows the percentage of identity, from lowest to highest, that each pair of genomes shares.

**Figure 2:**
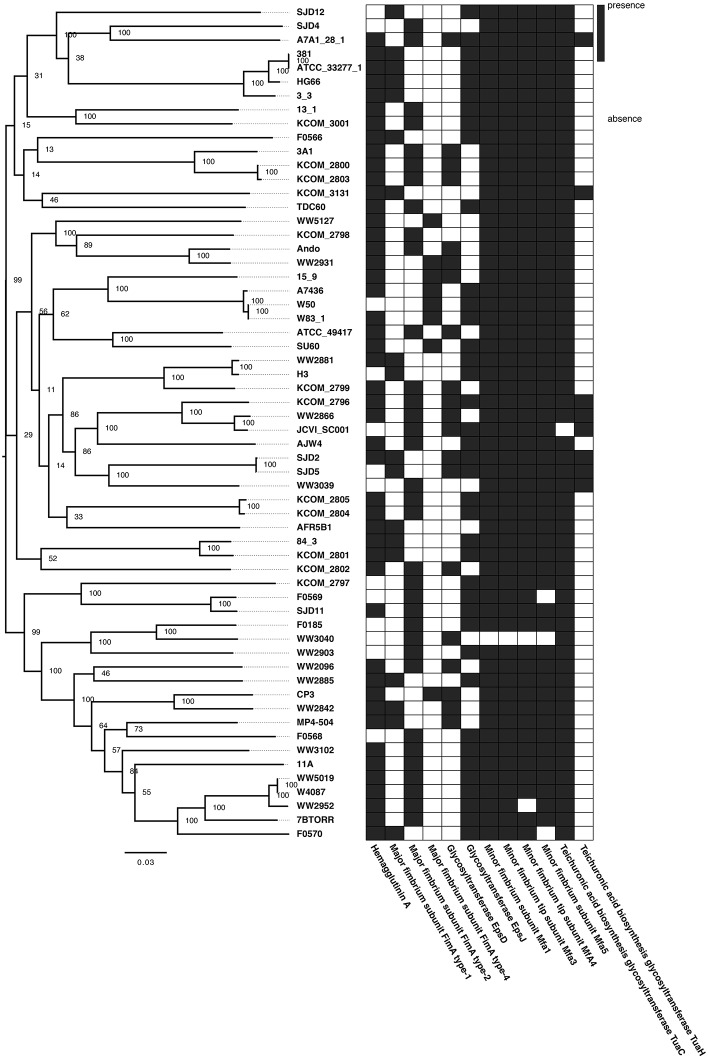
Phylogenetic relationships among *Porphyromonas gingivalis* strains and presence/absence of virulence genes. (Left) Maximum likelihood tree inferred from a core SNPs alignment (16,645 polymorphic positions) of the 64-genome *P. gingivalis* dataset. Node labels represent the bootstrap support values. (Right) Heatmap showing the presence (black) or absence (white) of virulence genes in each *P. gingivalis* genome (only virulence genes belonging to the accessory genome are shown).

In order to compare the whole-genome nucleotide identity of CP3 and H3 genomes with other previously sequenced *P. gingivalis* strains, an ANI analysis using the 64-genome *P. gingivalis* dataset (i.e., 62 genomes from RefSeq plus CP3 and H3 genomes) was performed (Table S3). The analysis shows high nucleotide identity among them (>98.22%; alignment fraction >80%; Figure 1). The strain WW2842 shows the highest nucleotide identity value with the CP3 genome (99.13%), while the H3 genome shows the highest identity with WW2881 and KCOM 2799 (99.69 and 99.20%, respectively; Table S4). There are no publications reporting virulence studies for WW2842, WW2881 or KCOM 2799 strains. However, WW2842 and WW2881 were isolated from subgingival plaque of patients with advanced periodontal disease in London, United Kingdom (BioProject: PRJNA401301, BioSample: SAMN07602651, BioSample: SAMN07602653). KCOM 2799 also was isolated from the subgingival plaque of a patient with periodontal disease in Gwangju, Korea (BioProject: PRJNA415884, BioSample: SAMN07836928). Moreover, the phylogenetic tree shows that CP3 and WW2842, and H3 and WW2881 and KCOM 2799 are grouped together, while CP3 and H3 (showing a 98.57% of identity), are found in different clades (Figure 2). The phylogeny also shows that CP3 is closer to the virulent strain MP4-504 (To et al., 2016), compared with other virulent strains such as W50, W83, ATCC49417, and A7436, which are more distantly related (Figure 2; Ebersole et al., 1995; Chen et al., 2004). On the other hand, H3 is phylogenetically related to AJW4, a previously reported very low invasive strain (Dolgilevich et al., 2011). However, H3 is found at a greater distance from other two type strains, ATCC 33277 and 381, which are comparatively less virulent than W50, W83 (Ebersole et al., 1995; Chen et al., 2004). These results suggest a heterogeneous nature of *P. gingivalis* species, not necessarily related to the periodontal status of their reservoirs.

From the virulence genes characterized for *P. gingivalis* in the literature (**Table 2**), 12 are part of the 64-genome dataset accessory genome and were selected to evaluate their distribution along the phylogeny (Figure 2). Results show that identical patterns of virulence factor presence and absence belong to phylogenetically unrelated and often geographically separated *P. gingivalis* strains that exhibit similar virulence phenotype. For instance, *P. gingivalis* 15_9 belongs to a clade with other virulent strains (W83; W50; A7436) and exhibits exactly the same pattern of virulence genes than CP3, though they are not closely related (Figure 2). Likewise, SJD12 strain exhibits the same pattern of virulence genes than H3 while being far apart in the phylogeny. Altogether, these results suggest that similar patterns of presence/absence of virulence genes may arise by convergent evolution and yield similar phenotypes.

### *P. gingivalis* Pan-Genome and Virulence Genes

Since the *P. gingivalis* strains included in these analyses come from different individuals and many of them are associated with different levels of virulence, a pan-genome analysis of the 64-genome *P. gingivalis* dataset was performed. The pan-genome is composed of 7,886 genes, of which 1,229 belong to the core genome and 6,657 genes belong to the accessory genome. Specifically, 65.44% of the CP3 genes and 64.08% of the H3 genes belong to the core genome, while 34.56% of the CP3 genes and 35.92% of the H3 genes belong to the accessory genome (Table 1).

**Table 1:** Pan-genome results of the 64-genome *Porphyromonas gingivalis* dataset.

**Strain**	**Total genes**	**Core genome**	**Accessory genome**
**CP3**	**1,878**	**65.44%**	**649 (34.56%)**
**H3**	**1,918**	**64.08%**	**689 (35.92%)**
ATCC_33277_1	1,907	64.45%	678 (35.55%)
ATCC_33277_2	1,940	63.35%	711 (36.65%)
W50	1,870	65.72%	641 (34.28%)
WW2842	1,854	66.29%	625 (33.71%)
WW2881	2,073	59.29%	844 (40.71%)
KCOM_2799	2,073	59.29%	844 (40.71%)
11A	1,919	64.04%	690 (35.96%)
13_1	1,970	62.39%	741 (37.61%)
15_9	1,863	65.97%	634 (34.03%)
381	1,944	63.22%	715 (36.78%)
3A1	1,965	62.54%	736 (37.46%)
3_3	1,907	64.45%	678 (35.55%)
7BTORR	1,868	65.79%	639 (34.21%)
84_3	1,966	62.51%	737 (37.49%)
A7436	1,974	62.26%	745 (37.74%)
A7A1_28_1	1,900	64.68%	671 (35.32%)
A7A1_28_2	1,861	66.04%	632 (33.96%)
AFR5B1	1,904	64.55%	675 (35.45%)
AJW4	1,971	62.35%	742 (37.65%)
ATCC_49417	2,092	58.75%	863 (41.25%)
Ando	1,847	66.54%	618 (33.46%)
F0185	1,876	65.51%	647 (34.49%)
F0566	1,926	63.81%	697 (36.19%)
F0568	1,975	62.23%	746 (37.77%)
F0569	1,872	65.65%	643 (34.35%)
F0570	1,935	63.51%	706 (36.49%)
HG66	1,989	61.79%	760 (38.21%)
JCVI_SC001	2,064	59.54%	835 (40.46%)
KCOM_2796	2,065	59.52%	836 (40.48%)
KCOM_2797	2,019	60.87%	790 (39.13%)
KCOM_2798	2,034	60.42%	805 (39.58%)
KCOM_2800	1,852	66.36%	623 (33.64%)
KCOM_2801	2,098	58.58%	869 (41.42%)
KCOM_2802	1,971	62.35%	742 (37.65%)
KCOM_2803	1,972	62.32%	743 (37.68%)
KCOM_2804	2,062	59.60%	833 (40.40%)
KCOM_2805	2,069	59.40%	840 (40.60%)
KCOM_3001	1,943	63.25%	714 (36.75%)
KCOM_3131	1,940	63.35%	711 (36.65%)
MP4-504	1,991	61.73%	762 (38.27%)
SJD11	1,938	63.42%	709 (36.58%)
SJD12	1,918	64.08%	689 (35.92%)
SJD2	2,006	61.27%	777 (38.73%)
SJD4	1,869	65.76%	640 (34.24%)
SJD5	1,946	63.16%	717 (36.84%)
SU60	1,893	64.92%	664 (35.08%)
TDC60	1,933	63.58%	704 (36.42%)
TDC_60	1,930	63.68%	701 (36.32%)
W4087	1,853	66.32%	624 (33.68%)
W83_1	1,953	62.93%	724 (37.07%)
W83_2	1,952	62.96%	723 (37.04%)
WW2096	1,958	62.77%	729 (37.23%)
WW2866	1,939	63.38%	710 (36.62%)
WW2885	1,998	61.51%	769 (38.49%)
WW2903	1,983	61.98%	754 (38.02%)
WW2931	1,935	63.51%	706 (36.49%)
WW2952	1,941	63.32%	712 (36.68%)
WW3039	1,944	63.22%	715 (36.78%)
WW3040	1,837	66.90%	608 (33.10%)
WW3102	1,903	64.58%	674 (35.42%)
WW5019	1,910	64.35%	681 (35.65%)
WW5127	1,976	62.20%	747 (37.80%)

*The table shows the total number of genes (“Total genes”), the percentage of genes that belong to the core genome (“Core genome”), and the number (and percentage) of accessory genes in each strain genome. Bold values correspond to the P. ginigavils strains CP3 and H3, whose genomes were sequenced and analyzed in this study*.

**Figure 3:**
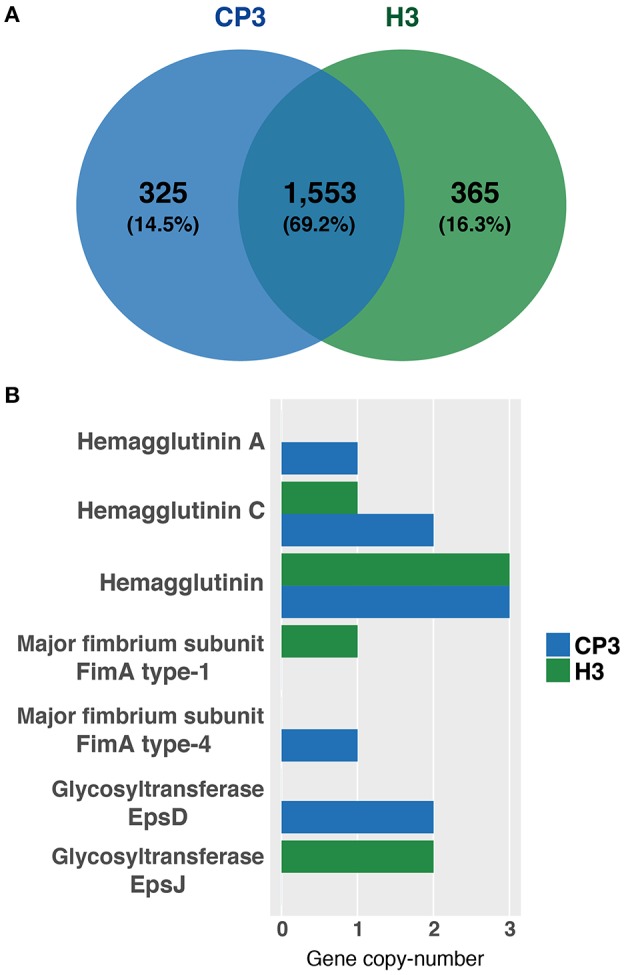
Common genes between *Porphyromonas gingivalis* CP3 and H3 and differences in the copy-number of virulence genes. **(A)** Venn diagram showing the number of common and unique genes of CP3 (blue) and H3 (green) genomes. Values in parentheses are the percentage of genes of the total genes in CP3 and H3 pan genome. **(B)** Bar plot showing six virulence genes that we detected to have differences in their copy-number between CP3 (blue bars) and H3 (green bars) genomes.

Next, we asked whether genes previously associated with virulence in *P. gingivalis* (Table 2) were either present or absent in both CP3 and H3 genomes. To this end, an exhaustive search of the virulence genes reported for *P. gingivalis* ATCC 33277 (reference strain) in the literature, and a screening of these genes in CP3 and H3 genomes through a bidirectional blast was conducted (Table 2). Interestingly, most of the virulence genes reported in the genome of the ATCC 33277 strain are also present in both CP3 and H3 genomes, including those encoding virulence factors as important as gingipains and capsule synthesis proteins (Table 2; Figure 2). Also, the pan-genome analysis was used to evaluate the presence/absence of virulence genes in CP3 and H3 genomes. Intriguingly, although both CP3 and H3 possess hemagglutinin genes, *hagA* and *hagC* are differentially found in their genomes. While CP3 have six ORFs coding for hemagglutinins (CP3_01418, CP3_01421, CP3_01422, CP3_01423, CP3_01568, CP3_01901), H3 have only four (H3_00216, H3_01383, H3_01678, H3_01679). Three of these genes belong to the CP3 and H3 core genome (>95% identity) and encode non-characterized hemagglutinins (CP3_01422, CP3_01423, CP3_01568; H3_00216, H3_01678, H3_01679). The other three *hag* copies found in CP3 genome and one of the H3 gene copies belong to their accessory genome (<95% identity). Moreover, there are differences in the type of hemagglutinins found in the accessory genome: while *hagA* is only present in CP3 (CP3_01901) and no in H3, CP3 possesses two copies of *hagC* (CP3_01418, CP3_01421), while H3 possesses only one (H3_01383) (Figure 3).

**Table 2:** Genes involved in *Porphyromonas gingivalis* virulence.

**Colonization steps and genes involved in** ***Porphyromonas gingivalis*** **virulence**								
**Virulence factor**	**Adherence /Invasion to epithelial cells**	**Cytokine production from epithelial cells**	**Antimicrobial peptides resistance**	**Biofilm formation and co-aggregation ability**	**PGN_1051 (O-antigen ligase)**	**P: Presence/A: Absent (locus)**		**References**
						**CP3**	**H3**	
Gingipains				PGN_0023 (hypothetical protein)		P (CP3_00770)	P (H3_00615)	Stathopoulou et al., 2009; Li and Collyer, 2011; Maisetta et al., 2011; Tancharoen et al., 2015
	PGN_1466 (*rgpB*)	PGN_1728 (*kgp*)	PGN_1466 (*rgpB*)	PGN_1466 (rgpB)		P (CP3_01530)	P (H3_00694)	
	PGN_1728 (*kgp*)		PGN_1728 (*kgp*)			A	A	
	PGN_1970 (rgpA)		PGN_1970 (rgpA)			P (CP3_01656)	P (H3_01892)	
Fimbria	PGN_0180 (*fimA*)	PGN_0180 (*fimA*)		PGN_0180 (*fimA*)		A	P (H3_01400)	Weinberg et al., 1997; Yamamoto et al., 2011
				PGN_0183 (*fimC*)		P (CP3_00162)	P (H3_01398)	
				PGN_0185 (*fimE*)		P (CP3_00164)	P (H3_01396)	
				PGN_0287 (*mfa1*)		P (CP3_00509)	P (H3_00313)	
				PGN_0288 (hypothetical protein)		P (CP3_00510)	P (H3_00314)	
Hemagglutinin A (HagA)	PGN_1733 (*hagA*)			PGN_1733 (*hagA*)		P (CP3_01901)	A	Yamamoto et al., 2011; Bélanger et al., 2012
				PGN_1906 (*hagC*)		P (CP3_01418, CP3_01421)	P (H3_01383)	
Fur	PGN_0465 (*Pgfur*)					P (CP3_01101)	P (H3_01350)	Ciuraszkiewicz et al., 2014
Hemin-binding protein 35 (HBP35)	PGN_0615 (*hbp35*)					A	A	Hiratsuka et al., 2010
Heat-stress proteins HtR and ClpB	PGN_0593 (*htrA*)					A	A	Yuan et al., 2007, 2008; Hiratsuka et al., 2010
	PGN_1118 (*clpB*)					P (CP3_01657)	P (H3_00984)	
Glycosyltransferase (GtfA)	PGN_0750 (*gtfA*)					P (CP3_00291)	P (H3_00666)	Narimatsu et al., 2004
Diguanylate cyclase	PGN_1932					A	A	Chaudhuri et al., 2014
SerB	PGN_0653	PGN_0653				P (CP3_00234)	P (H3_01482)	Hasegawa et al., 2008
Capsule		capsular polysaccharide biosynthesis loci PGN_0106-PGN_0120	capsular polysaccharide biosynthesis loci PGN_0106-PGN_0120			P (CP3_00200, CP3_00202 - CP3_00207)	P (H3_01649 - H3_01654, H3_01884)	Chen et al., 2004; Aduse-Opoku et al., 2006; Yamamoto et al., 2011
				PGN_1100 (Putative capsule biosynthesis protein CapA)		P (CP3_01628)	P (H3_01830)	
LPS		PGN_1051 (O-antigen ligase)	PGN_1051 (O-antigen ligase)		PGN_1051 (O-antigen ligase)	A	A	Coats et al., 2009; Yamamoto et al., 2011; Díaz et al., 2015
			PGN_0525 (Lipid A 4′-Phosphatase)			P (CP3_01077)	P (H3_00138)	
				PGN_0206 (Putative lipid A disaccharide synthase)		P (CP3_00187)	P (H3_01701)	
				PGN_0376 (2-Dehydro-3-deoxyphosphooctonate aldolase)		P (CP3_01045)	P (H3_01027)	
				PGN_0544 (3-Deoxy-D-manno-octulosonic acid transferase)		P (CP3_01752)	P (H3_00118)	
				PGN_0696 (Probable hydrolase)		P (CP3_00238)	P (H3_01478)	
				PGN_0777 (Probable glycosyl transferase)		P (CP3_01389)	P (H3_01443)	
				PGN_1054 (*vimF*)		P (CP3_00990)	P (H3_01148)	
				PGN_1235 (*porS*)		P (CP3_01338)	P (H3_01005)	
				PGN_1239 (Probable lipopolysaccharide biosynthesisglycosyltransferase)		P (CP3_01341)	P (H3_01008)	
				PGN_1255 (Putative heptosyltransferase)		P (CP3_01354)	P (H3_01957)	
				PGN_1310 (Glycogen synthase)		P (CP3_00763)	P (H3_00607)	
				PGN_1614 (UDP-glucose 4-epimerase)		P (CP3_00072)	P (H3_00074)	
				PGN_1718 (Probable UDP-2,3-diacylglucosamine hydrolase)		P (CP3_01706)	P (H3_00375)	
				PGN_1750 (Putative 3-deoxy-D-mannooctulosonatecytidylyltransferase)		P (CP3_01505)	P (H3_00808)	
				PGN_2018 (Putative UDP-N-acetylglucosamineacyltransferase)		P (CP3_00538)	P (H3_01067)	
				PGN_2019 (UDP-3-O-_3-hydroxymyristoyl_ Nacetylglucosaminedeacetylase)		P (CP3_00537)	P (H3_01066)	
				PGN_2020 (UDP-3-O-_3-hydroxymyristoyl_ glucosamine N-acyltransferase)		P (CP3_00536)	P (H3_01065)	

Presence/absence in CP3 and H3 genomes of genes associated with virulence, previously reported in the literature for P. gingivalis. The first six columns show a classification of these genes, indicating the corresponding locus in the P. gingivalis reference strain ATCC 33277 (PGN_^*^) along with the gene name (in parentheses). The following two columns show the presence (P)/absence (A) of these genes in CP3 and H3 genomes, along with their respective locus (in parentheses). Finally, the next column contains the reference in which those genes were found to be associated with virulence in P. gingivalis.

Another interesting virulence-associated gene is *fimA*, which encodes different major fimbriae types. Interestingly, while type IV *fimA* is present in CP3 and absent in H3, type I *fimA* is present in H3 and absent in CP3 (Figures 2, 3). Finally, differences in two other genes were detected between both strains; while *epsD* gene encoding for a putative glycosyltransferase was only present in CP3, *epsJ* was found in H3 and not in CP3 (Figures 2, 3).

### Hemagglutination Ability of CP3 and H3 Strains

**Figure 4:**
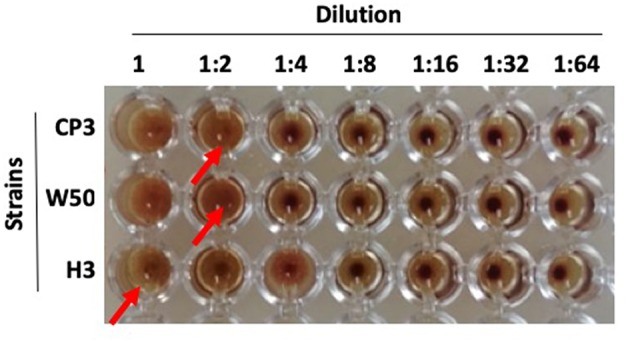
Hemagglutination activity of *Porphyromonas gingivalis* strains CP3, H3, and W50. Hemagglutinin activities of *P. gingivalis* (W50, H3, and CP3) were assessed in cells serially diluted (from left to right) and incubated with sheep erythrocytes (2%) in a round-bottom microtiter plate. Dilution folds were listed on the top. Red arrows indicate the last dilution that showed full agglutination. The figure is representative of three biological replicates.

As described above, the CP3 strain possesses one copy of *hagA* and two of *hagC*, while H3 possesses only one copy of *hagC* (Figure 3). In order to evaluate whether there were differences in the hemmaglutination ability between CP3 and H3 that could be associated with these genetic differences, an *in vitro* hemagglutination assay was performed. Our results show that while CP3 was able to agglutinate red cells until dilution 1:2 (1.5 × 10^8^ CFUs), H3 only agglutinates when 3.0 × 10^8^ CFUs where added. The hemagglutination titer of red blood cells showed by the virulent reference strain W50 was similar compared to CP3 and higher compared to H3 (Figure 4). Therefore, although H3 hemagglutinate red cells, it requires a higher bacterial load compared to CP3 and W50. In order to evaluate if the differences in the copy number of the *hagC* gene could influence the hemagglutination ability of H3 and CP3, we evaluated its expression by RT-qPCR. Accordingly, the expression of *hagC* was significantly lower in H3 strain compared to CP3, which suggests that the copy number might explain the observed differences in hemagglutination (Figure S1). In addition, we evaluated the *hagC* transcript levels of the reference strain W50, determining a higher expression compared to H3.

### Adherence and Biofilm Formation Abilities of CP3 and H3 Strains

**Figure 5:**
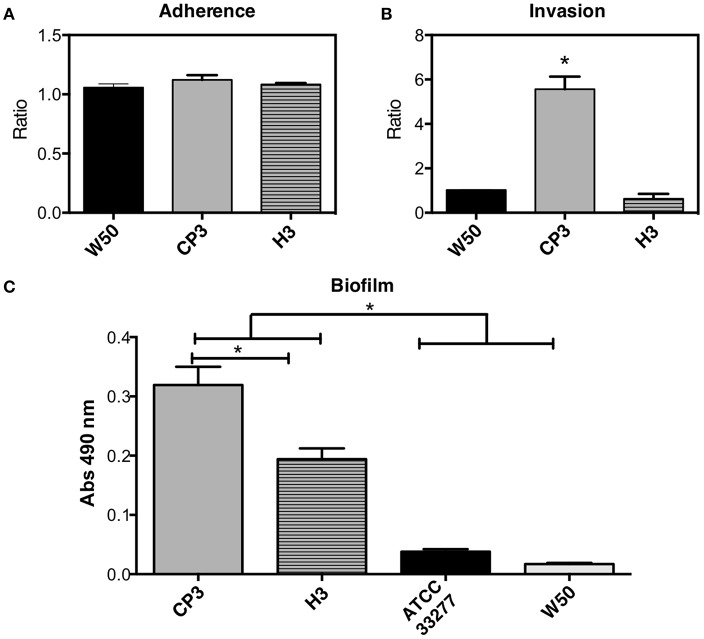
Cellular adherence **(A)**, invasion **(B)**, and biofilm formation **(C)** of different strains of *Porphyromonas gingivalis* exhibiting different *fimA* genotypes. Adherence **(A)** or invasion **(B)** were calculated as: the number of viable CFU in infected cells/the number of CFU of initial bacteria inoculum. Biofilm formation **(C)** was determined by safranin staining. The dye retained in the biofilm was eluted and measured at 490 nm. Invasion, adherence and biofilm assays were performed in triplicate on at least three independent occasions. Values that differ significantly from controls are indicated (SEM; *n* = 3; ^*^p ≤ 0.05).

Since CP3 possesses fimbria type IV (widely associated with epithelial cell invasion) and H3 possesses fimbria type I (found in the periodontal healthy population; Amano et al., 2000), an assay was performed to evaluate if such genetic differences are reflected in their adhesion and invasion ability to oral epithelial cells. Although no differences in attachment level to epithelial cells were observed between H3, CP3 and the W50 reference strain (Figure 5A), CP3 invasion ability was ~6-fold higher than both H3 and the reference strain (Figure 5B).

Since there are differences in the *fimA* genes encoded in CP3 and H3 genomes, the next step was to evaluate their biofilm formation ability. Results show that CP3 (encoding type IV *fimA*) possesses a significantly higher ability to form biofilms that H3 (encoding type I *fimA*) (Figure 5C). Interestingly, both strains formed more biofilm than both reference strains ATCC 33277 (type I) and W50 (type IV).

## Discussion

*P. gingivalis* has been largely associated with periodontitis, showing both one of the highest prevalence and abundance among periodontitis-associated species (Griffen et al., 2012; Abusleme et al., 2013; Kistler et al., 2013; Hong et al., 2015; Kirst et al., 2015; Park et al., 2015). Nevertheless, using molecular approaches, *P. gingivalis* have also been detected in periodontally healthy individuals, although in lower abundance (Abusleme et al., 2013; Díaz et al., 2015; Choi et al., 2018).

We previously studied two *P. gingivalis* clinical isolates showing differences in their virulence, one from a chronic severe periodontitis patient (CP3), and the other from a periodontally healthy subject (H3) (Díaz et al., 2015; Soto et al., 2016). Interestingly, virulent properties of these strains are in agreement with the clinical signs linked to periodontal damage determined in the donor subjects. Periodontal parameters observed in the subject from whom the CP3 isolate was obtained had an average probing depth (PD) = 4.2 mm and a clinical attachment loss (CAL) = 6.1 mm. Moreover, from the 107 sites analyzed in the oral exam, 47 sites were ≥ 5 mm and 13 sites ≥ 7 mm, indicating a high level of tissue damage (Table S1). In contrast, the periodontally healthy subject (from whom H3 was obtained) had a PD = 2.5 mm and a CAL = 3.6 mm. No sites ≥ 5 mm were observed from 167 sites analyzed, 166 were ≤ 3 mm (Table S1).

In previous works, we showed that CP3 –compared with H3- showed both higher resistance to antimicrobial peptides (Díaz et al., 2015) and ability of decreasing apoptosis of gingival epithelial cells (Soto et al., 2016). A phenotype that was associated with the presence of high molecular O-antigen molecules in CP3 (Díaz et al., 2015). Therefore, in this work we investigated the existence of other genotypic features that could explain differences in virulence that help us to understand the contribution of *P. gingivalis* to periodontitis.

Our results showed that CP3 and H3 genomes share ~98 to ~99% identity with other available *P. gingivalis* genomes with an alignment coverage from ~83 to 95%; this translates in a ~64 to 65% of common genes (core genome; >95% identity) and ~34 to 35% of “unique” genes (accessory genome; <95% identity; Figure 1). Although both strains locate in relatively distal branches in the phylogenetic tree, they are not necessarily closer to either more virulent strains (in the case of CP3), or to less virulent ones (in the case of H3). However, relevant genes involved in virulence show different patterns of presence/absence among *P. gingivalis* strains (Figure 2).

Notably, we were able to correlate genomic differences with virulence features. First, CP3, harboring one copy of the hemagglutinin gene *hagA* and two copies of *hagC*, shows higher hemmaglutination ability *in vitro* compared to H3 (Figures 3, 4), which encodes only one copy of *hagC* gene (Figure 3). HagA has been widely related erythrocytes adhesion for nutrients acquisition (Lépine and Progulske-Fox, 1996; Lamont and Jenkinson, 2000). Therefore, we speculate that its absence in H3 impairs its hemagglutination ability. Variation of comparable magnitude in the hemagglutination ability of *P. gingivalis* strains has been previously reported in strains with other genetic backgrounds (Shoji et al., 2013; Zhang et al., 2016; Puth et al., 2019). Interestingly, among *P. gingivalis* hemagglutinins, *hagA, hagD*, and *hagE* show >70% identity, while *hagB* and *hagC* correspond to unrelated families, sharing 93% identity (Connolly et al., 2017). Moreover, although HagB and HagC functions have only been partially defined, it has been reported that both of them produce hemagglutination, although they are not the sole responsible for such activity (Lépine and Progulske-Fox, 1996). Interestingly, hemagglutination in *P. gingivalis* has been associated to other proteins, such as specific domains of gingipains RgpA and Kgp (Li et al., 2011). Also, it was recently reported that *pckA* contributes to hemagglutination, independently of the gingipains activity (Wu et al., 2018). Thus, in spite of an association between the genomic differences in *hagA* and *hagC*, as well as the levels of expression of hagC (Figure S1) with the hemagglutination activity found in this study, the presence of other(s) virulence factor(s) of *P. gingivalis* that also contribute to this phenotype cannot be discarded.

To our knowledge, no evidence of association between virulence and copy numbers of specific genes has been described in *P. gingivalis*. However, in another bacterial model, no association between the virulence of *Vibrio parahaemolyticus* and the number of copies of Vp_PirA- and B-like toxin genes in their genome was found (Pérez-Chaparro et al., 2008). In this context, the question of whether the sole copy of *hagC* found in H3 is either not functional or not sufficient to produce hemagglutination remains to be elucidated. Nevertheless, our results show that a H3 exhibit a lower expression of *hagC* compared to CP3, which is associated with a lower hemagglutination capacity of H3 strain, suggesting a functional role for this difference.

Similarly, others have also reported genetic differences that rely not only in presence/absence of specific genes. Several studies have reported extensive genetic heterogeneity among *P. gingivalis* strains obtained from periodontitis patients, using molecular typing methods such as restriction endonuclease analysis (REA) (Genco and Loos, 1991), restriction fragment length polymorphism (RFLP) (Loos and Dyer, 1992), multilocus enzyme electrophoresis (MEE) (Loos et al., 1993), and arbitrarily primed polymerase chain reaction (AP-PCR) (Ménard et al., 1992). Furthermore, variations in the DNA of *P. gingivalis* analyzed by heteroduplex assays [that permit to identify polymorphisms in the ribosomal internal spacer region (ISR)], revealed the presence of heteroduplex types; some of them significantly associated with periodontitis and others equally detected in health or disease (Griffen et al., 1999).

Regarding FimA genotypes, type I *fimA* has been found mainly in the periodontally healthy population (Feng et al., 2014), as well as in the avirulent type-strain ATCC 33277. In turn, type IV *fimA* is more frequently associated with *P. gingivalis* obtained from periodontitis patients (Amano et al., 2004) and is also present in the virulent reference strain W83 (Figure 2). Moreover, it has been shown that phenotypes based on the presence/absence of the six variants of nucleotide sequences of *fimA* gene (types I to VI) induce different cellular responses (Missailidis et al., 2004). Particularly, type I FimA have been associated with biofilms with a dense basal monolayer and disperse microcolonies; whereas type IV FimA, with thicker biofilms (Kuboniwa et al., 2009). This is consistent with our results: H3 lacks gene CP3_00160 coding for the major fimbrium subunit FimA type IV, but exhibits a major fimbrium subunit FimA type I (Figure 3). In contrast, CP3 lacks the gene coding for major fimbrium subunit FimA type I and possesses the gene coding for the major fimbrium subunit FimA type IV. This genotypic difference is associated with the lower biofilm formation ability of H3, as well as with less intracellular invasion to oral epithelial cells compared to the CP3 strain (Figure 5).

Interestingly, among *P. gingivalis* virulence factors, not only FimA has been related to epithelial cell adhesion/invasion and biofilm formation. HagA promotes adhesion to epithelial cells, and antibodies against its hemagglutinin domain protects against oral infection in a murine model (Frazer et al., 2006; Bélanger et al., 2012). Therefore, it cannot be discarded that the differences in the hemagglutinins encoded in CP3 genome, and no in H3, are also contributing to CP3 adhesion/invasion abilities in our *in vitro* model. The *hagB* gene of *P. gingivalis*, which is 98.6% identical to *hagC*, encodes HagB that mediates adhesion to host cells (Progulske-Fox et al., 1999; Song et al., 2005). Hence, it is likely that one could “substitute” the other's function. Therefore, as discussed in the context of differences in hemagglutination, it is unlikely that the lack in only one copy of *hagC* found in H3 has a major effect on its ability of adhere to and invade epithelial cells. Similarly, HagA has been associated with co-aggregation between *P. gingivalis* and *Treponema denticola*, which is another periodontitis-associated bacteria (Ito et al., 2010); and HagC mediates multi-species biofilm formation (Connolly et al., 2017). Thus, besides FimA, CP3 enhanced ability to form biofilms could also be attributed to hemagglutinin differences compared to H3.

Unlike previous reports comparing clinical isolates obtained from patients showing different periodontal disease severity, no differences in capsular antigen genotypes were found between CP3 and H3 strains (van Winkelhoff et al., 1993; Laine et al., 1996; Laine and van Winkelhoff, 1998). Such differences have also been found between the reference strains W83 and ATCC 33277; while the gene coding for the synthesis of capsular polysaccharide is present in W83, it is absent in the ATCC 33277 (Chen et al., 2004). Nonetheless, while the glycosyltransferase EpsD was only present in CP3, the glycosyltransferase EpsJ was only found in H3 (Figures 2, 3). Although, no virulence function has been attributed to such proteins in *P. gingivalis*, they have been related to exopolysaccharide synthesis in other bacterial species. In *Bacillus subtilis*, the locus *epsHIJK* is necessary for the synthesis poly-*N*-acetylglucosamine, which is a key component of the biofilm (Roux et al., 2015). *epsJ* genes are annotated as putative glycosyltransferases, showing similarity to *icaA* in *Staphylococcus aureus* and *pgaC* in *Escherichia coli*. EpsD and EpsJ have also been associated to exopolysaccharides synthesis in *Lactococcus lactis* (van Kranenburg et al., 1999). While EpsD links glucose-1-phosphate from UDP-glucose to a lipid carrier, EpsJ is likely to be involved in releasing the trisaccharide backbone from the lipid carrier. Due to the relevance of exopolysaccharides in biofilm formation and taking in to account the relevance of such structures in *P. gingivalis* virulence, we cannot discard that these genetic differences between CP3 and H3 could participate in their biofilm formation ability (Figure 4). Altogether, through the analysis of a dataset composed by 64 *P. gingivalis* genomes, this study provides relevant results regarding genetic determinants and their association with *P. gingivalis* virulence.

## Data Availability

The datasets generated for this study can be found in NCBI PRJNA521311.

## Author Contributions

KM performed the comparative genomics analyses, wrote, and reviewed the manuscript. AH analyzed the data, wrote and reviewed the manuscript. CS carried out hemagglutination, adhesion/invasion assays, and RT-qPCR experiments. IB carried out biofilm assays. MO carried out hemagglutination assays. CM performed sequencing of *P. gingivalis* strains. JP-D contributed to conception and critically reviewed the manuscript. EC-N contributed to the design of the genomic analyses, wrote, and reviewed the manuscript. DB conceived and designed the research, wrote, and reviewed the manuscript. All authors contributed to the final version.

### Conflict of Interest Statement

The authors declare that the research was conducted in the absence of any commercial or financial relationships that could be construed as a potential conflict of interest.

## Supplementary Material

Table S1 Periodontal parameters of the subjects from whom *Porphyromonas gingivalis* strains CP3 and H3 were obtained. PD, pocket depth; CAL, clinical attachment level.

Table S2 Sequencing, quality control (QC) of reads, and assembly of the *Porphyromonas gingivalis* strains CP3 and H3 genomes. The first panel “Sequencing and quality control (QC)” shows the number of sequenced reads and the number of reads after QC. The quality-controlled reads were used for *de novo* assembly of CP3 and H3 genomes. Statistical features of both assembled genomes are shown in the second panel “Genome assembly,” including the number of predicted proteins and the presence of bacterial orthologs (“Proteome completeness”). C, complete orthologs; S, complete and single-copy orthologs; D, complete and duplicated orthologs; F, fragmented orthologs; M, missing orthologs.

Table S3 Publicly available *Porphyromonas gingivalis* genomes used in this study. Sixty-two *P. gingivalis* genomes were downloaded from NCBI's RefSeq database in February 2018.

Table S4 Average nucleotide identity and coverage values for the 64-genome *Porphyromonas gingivalis* dataset. Sheets 1, 2 show the average nucleotide identity and coverage values, respectively. Sheets 3, 4 detail the identity and coverage values, respectively, of all *P. gingivalis* strain genomes against CP3 and H3 genomes.
