# Supplementary figures and images for: Variability in Genomic and Virulent Properties of Porphyromonas gingivalis Strains Isolated From Healthy and Severe Chronic Periodontitis Individuals
Source: Front Cell Infect Microbiol. 2019 Jul 10;9:246. doi: 10.3389/fcimb.2019.00246 (PMC6635597; doi:10.3389/fcimb.2019.00246)

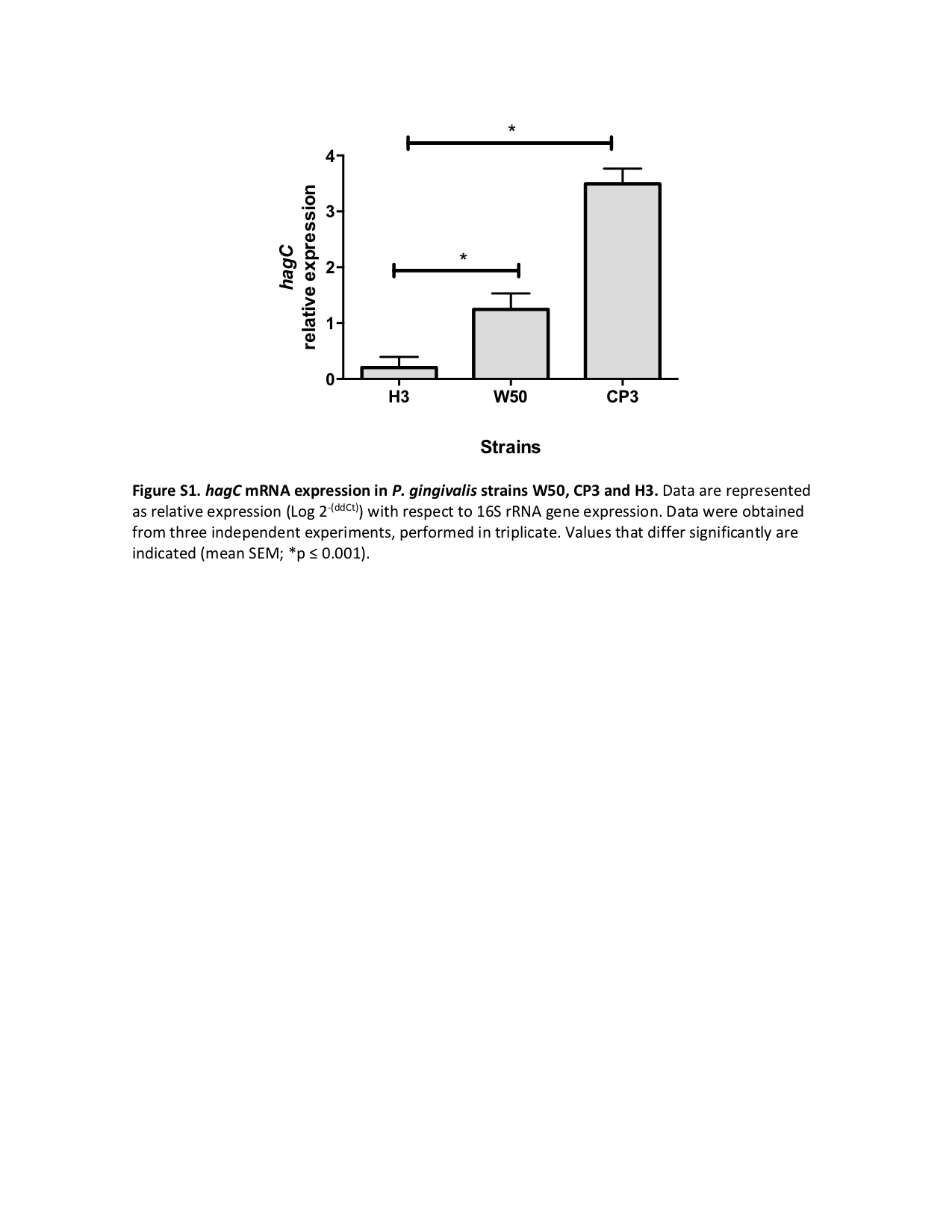

Supplement: Supplementary file 1 [file Image_1.TIFF]
